# Assessing Botanical Authenticity of Honey Through DNA-Metabarcoding-Based Pollen Analysis: A Preliminary Study

**DOI:** 10.3390/foods15193542

**Published:** 2026-10-04

**Authors:** Lucia Ranieri, Roberta Piredda, Anna Mottola, Chiara Intermite, Martina Denami, Lucilia Lorusso, Angela Di Pinto

**Affiliations:** 1 Department of Veterinary Medicine, University of Bari Aldo Moro, Prov. le Casamassima 62, km 3, 70010 Valenzano (Bari), Italy; lucia.ranieri@uniba.it (L.R.); robpiredda@gmail.com (R.P.); c.intermite2@phd.uniba.it (C.I.); lucilia.lorusso15@gmail.com (L.L.); angela.dipinto@uniba.it (A.D.P.); 2 Marine Organism Taxonomy Core Facility (MOTax), Stazione Zoologica Anton Dohrn, Villa Comunale, 80121 Naples, Italy; 3 Laboratory of Palynology and Palaeobotany, Department of Life Sciences, University of Modena and Reggio Emilia, 41125 Modena, Italy; 336339@studenti.unimore.it

**Keywords:** botanical authenticity, honey, DNA metabarcoding, melissopalynology

## Abstract

Honey is highly vulnerable to botanical mislabeling and fraud, highlighting the need for dedicated analytical approaches capable of reliably supporting authenticity assessment. To the best of our knowledge, this study is the first to integrate traditional melissopalynology and *rbcL* DNA metabarcoding for the botanical characterization of honeys collected from Apulian beekeepers. Melissopalynological analysis confirmed the labeled floral source in 6/10 (60%) samples, while four samples were reclassified. One cherry honey was identified as polyfloral, and one robinia (acacia) honey as crimson clover honey. The two samples labeled as polyfloral showed a predominance of chestnut and coriander pollen. In contrast, molecular results confirmed label declarations for chestnut and cherry honeys, whereas robinia (acacia) honeys highlighted known limitations associated with under-represented pollen taxa. In terms of biodiversity, DNA metabarcoding identified 128 plant families, whereas melissopalynology detected only 42. Despite this difference in taxonomic resolution, both approaches converged on the core botanical composition of the honey matrix, sharing 39 plant families that represent the principal nectariferous and entomophilous taxa. Overall, melissopalynology proved decisive for regulatory label verification, while DNA metabarcoding provided a broader qualitative description of the botanical composition. As a preliminary pilot study, the combined application of melissopalynology and DNA metabarcoding may contribute to a more comprehensive assessment of honey botanical origin and traceability; however, molecular detection alone cannot be considered sufficient to establish compliance with legal requirements for unifloral honey labeling.

## 1. Introduction

Honey is defined in the Codex Alimentarius, published by the Food and Agriculture Organization (FAO) of the United Nations, as “the natural sweet substance produced by honey bees from nectar of blossoms or from secretions of living parts of plants or excretions of plant-sucking insects on the living parts of plants, which honey bees collect, transform and combine with specific substances of their own, store and leave in the honeycomb to ripen and mature” [1]. Given its natural compositional features, honey remains one of the most frequently adulterated food products worldwide, presenting a major challenge for food safety authorities and the global food industry [2]. Common fraudulent practices include the addition of sugar syrups or dilution with lower-quality honeys, and mislabeling of botanical or geographical origin, all of which compromise consumer trust and market sustainability [3]. Honey may be classified as unifloral when its nectar is predominantly derived from a single botanical source, whereas polyfloral honey originates from multiple floral sources. Because unifloral honeys generally command higher market prices than polyfloral honeys [4,5,6,7], reliable botanical characterization is relevant not only for verifying label compliance but also for supporting product differentiation and valorization. To address these risks, Regulation (EU) No. 1169/2011 establishes general food labeling requirements, while Directive 2001/110/EC specifically regulates honey labeling, including the declaration of country and, under defined conditions, botanical origin. More recently, Directive (EU) 2024/1438 has strengthened origin labeling for honey blends and promoted harmonized analytical approaches for authenticity verification. However, EU legislation does not prescribe specific methods for verifying botanical origin, highlighting the need for complementary analytical approaches such as melissopalynology [8], chromatographic and spectroscopic analysis [9], metabolomics [10] and DNA-based methods [11].

Considering the growing economic impact of honey adulteration [2,12], improving traceability through innovative authentication approaches represents a key step toward strengthening regulatory control and consumer protection [12,13]. To date, analysis of pollen content in honey (melissopalynology) represents the most widely accepted reference approach for the assessment of botanical and geographical origin, being recommended by technical bodies such as the International Honey Commission and referenced in Codex Alimentarius guidelines. In the Italian regulatory framework, this central role is further consolidated by the Ministerial Decree (D.M.) of 25 July 2003, which includes melissopalynology among the main official analyses for the characterization of honey [14]. Pollen analysis provides valuable information on the botanical composition of honey through microscopic identification of pollen grains. Honey is generally considered to be produced mainly from one plant (unifloral), where the pollen frequency of that plant is more than 45% [15,16]. However, due to multiple sources of pollen within the honey matrix, plus other complex botanical aspects (nectar, nectarless, and wind-blooming plants), a direct proportion between the nectar contribution of a plant to the composition of honey and the quantity of its pollen in the honey matrix is not possible [8,17]. Indeed, some pollen types in honey are over-represented, meaning that pollens appear in disproportionately high amounts in a honey matrix compared to the actual amount of nectar/pollen collected from those plants (i.e., *Myosotis* spp., *Castanea sativa*, and *Eucalyptus* spp.). Otherwise, pollen types in honey can be under-represented when the actual amount of nectar collected (e.g., *Thymus* spp., *Citrus* spp., and *Robinia* spp.) appears in much lower proportions in the final honey [15]. Consequently, pollen abundance does not necessarily reflect the relative contribution of a plant to the nectar source, and botanical characterization requires consideration of the characteristic representation of individual pollen taxa.

Given the intrinsic complexity of the honey matrix, specific characterization criteria have been developed for some unifloral honeys. In particular, UNI standards [18,19,20,21,22] and dedicated characterization sheets [6,23] have been established for some of the most relevant monofloral honeys (e.g., chestnut, acacia, citrus, eucalyptus, and honeydew honeys), assessing melissopalynological, physicochemical and sensory parameters to provide a more comprehensive definition of their botanical identity. For unifloral honeys for which no specific standards or characterization guidelines are available, the assessment of botanical origin is commonly based on the conventional criterion of a dominant pollen frequency exceeding 45%. Despite the key role played by melissopalynology, it is a time-consuming, operator-dependent method often limited to genus-level identification, with rare species-level resolution [24] due to the morphological similarities of pollen grains among closely related taxa. Moreover, their heterogeneous spatial distribution within the honey matrix further complicates accurate and reproducible analyses [25].

These intrinsic limitations of melissopalynology have stimulated the development of complementary molecular approaches capable of identifying botanical origin, independently of pollen morphology [2,26,27,28]. Chromatographic and spectroscopic approaches further support botanical origin verification by providing information ranging from detailed chemical fingerprinting to rapid screening. LC-MS and NMR-based untargeted metabolomics enable detailed fingerprinting of sugars, phenolics, and other metabolites to discriminate monofloral honeys [9], although these techniques remain costly and require specialized instrumentation and expertise, limiting their applicability for routine analysis. GC-MS characterizes volatile compounds linked to specific floral sources [9] but is constrained by lengthy sample preparation and sensitivity to seasonal variation in volatile profiles. FTIR and NIR spectroscopy offer faster, non-destructive alternatives suited to routine screening [10], though at the cost of lower discriminatory power and a strong dependence on large, representative reference databases for reliable calibration. More recently, DNA metabarcoding has emerged as a promising strategy for honey botanical profiling [29], enabling the simultaneous detection of multiple plant taxa through high-throughput sequencing of standardized barcode regions [2,30,31]. However, despite its extreme sensitivity and high taxonomic resolution, DNA metabarcoding is not without limitations. The method is inherently semi-quantitative, highly susceptible to taxa-specific PCR amplification biases, and influenced by differential DNA extraction efficiencies linked to pollen exine structure. Furthermore, its ability to capture broad environmental DNA (e-DNA) requires careful interpretation to distinguish primary nectar sources from secondary airborne background noise [29,32]. In addition, the molecular region used as plant DNA barcodes could affect the final botanical profile. Among several markers, such as *matK*, *trnL* (UAA), etc., the chloroplast *rbcL* region generally provides lower species-level resolution compared to nuclear markers (e.g., *ITS2*), but it is officially recommended by the CBOL Plant Working Group due to its high amplification success, ease of recovery from degraded matrices, and broad universality across land plants, making it a robust foundational marker for preliminary botanical surveys [33,34].

Although honey production represents an important component of the agri-food sector in the Apulia region, where favorable climatic conditions, rich floral biodiversity and traditional beekeeping practices support both unifloral and polyfloral honeys of high quality, this region is one of the least studied in terms of honey production and characterization [35]. To date, Apulian honeys have mainly been characterized using chemical and microscopic approaches [35,36]. However, molecular approaches for the characterization of their complex botanical composition remain poorly explored. To the best of our knowledge, no previous study has integrated DNA metabarcoding and melissopalynology for the characterization of Apulian honeys. Beyond its regional focus, this research addresses a critical methodological gap: evaluating how the integration of highly sensitive molecular tools with traditional morphological methods can resolve analytical ambiguities, particularly in complex matrices (e.g., masked polyfloral honeys) or in the presence of severely under- or over-represented pollen. In this context, the present study aims to provide a preliminary characterization of honeys produced by Apulian beekeepers by comparing DNA metabarcoding outcomes with traditional melissopalynological analysis. Specifically, this preliminary study aims to (i) characterize the botanical composition of the investigated honeys using both approaches, (ii) evaluate their degree of concordance and complementarity, and (iii) assess whether their integration may provide additional information relevant to the verification of declared botanical origin and honey traceability. By delineating botanical profiles and assessing their congruence with declared commercial origins, this work explores the potential of this integrated approach to support honey authenticity verification, traceability systems, and risk-based food safety monitoring.

## 2. Materials and Methods

### 2.1. Sampling

Between June and July 2023, ten honey samples bearing labels declaring different botanical origins were purchased directly from independent beekeepers located in different geographic areas across the Apulia region. The set included robinia (acacia) (*n* = 2), citrus (*n* = 2), chestnut (*n* = 2), cherry (*n* = 2) and polyfloral honey (*n* = 2). All samples were stored at room temperature in complete darkness until analysis (Table 1).

### 2.2. Labeling Analysis

Product labels were evaluated for compliance with Regulation (EU) No. 1169/2011 and Legislative Decree No. 179/2004. Each label was checked for mandatory and voluntary information, including product name, producer or packager name and address, production/packaging site (where different), country of origin, net weight, batch number, minimum durability date and botanical origin.

### 2.3. Melissopalynological Analysis

Melissopalynological analysis was performed to investigate the pollen spectrum of the honey samples and to verify their declared botanical origin through both qualitative and quantitative evaluations. The standard procedure is based on the microscopic examination of the sediment obtained from a honey–water solution after centrifugation and allows for the morphological identification and the quantification of pollen grains and other microscopic elements present in honey.

For each honey jar, approximately 100 mL was sent to an external specialized laboratory (Piana Ricerca e Consulenza srl, Castel San Pietro Terme, Italy) that performed the analysis according to the UNI 11299:2008 standard method. Sample preparation followed the protocol originally described by Louveaux et al. [15] and subsequently harmonized by Von der Ohe et al. [8], with minor modifications. Briefly, about 12 g of honey (instead of the standard 10 g, in order to ensure an adequate pollen concentration) was weighed and dissolved in previously heated distilled water. The solution was centrifuged at 3500 rpm (corresponding to a relative centrifugal force of 2068× *g*) for 10 min, and the obtained sediment was resuspended and centrifuged again for an additional 10 min. The recovered sediment was spread onto a microscopic slide and dried on a hotplate (40 °C). The dried sediment was covered with an appropriate coverslip using a drop of melted glycerin jelly as embedding medium. *Lycopodium* spore tablets were added before the treatment as an internal standard for the quantitative analysis. *Lycopodium* spore tablets were previously dissolved in 0.5 mL of concentrated hydrochloric acid (37%).

Microscopic identification was performed at 1000× magnification. For each sample, at least 300 pollen grains were counted, as required by the UNI 11299:2008 standard for frequency estimation. Taxonomic identification was supported by atlases and the specialized reference literature [37,38,39], as well as by comparison with the laboratory reference collection and database of honey pollen spectra from different geographical areas. Pollen grains were morphologically identified to species level whenever possible, otherwise mainly to genus or family level, depending on the diagnostic resolution.

Results are expressed as the relative percentage of each pollen type with respect to the total pollen count, and as percentages calculated considering only nectariferous taxa. Nectariferous, non-nectariferous and over-represented taxa were classified according to the reference lists reported by Persano Oddo et al. [35] and Ricciardelli D’Albore [38]. Pollen types from anemophilous species were also recorded, as they may provide information on the geographical origin of the samples. When a pollen type was detected outside the count or represented less than 1% of the total, it was recorded as 0.001. Honeydew elements (HDEs) were also counted, and other elements of the sediment (i.e., yeasts, starch grains, foreign particles) were recorded if present. Quantitative analysis allowed for the estimation of the absolute number of pollen grains per 10 g of honey (GP/10 g) and the calculation of the honeydew element-to-pollen ratio (HDE/P).

### 2.4. DNA Extraction, Amplification, and Sequencing

Prior to DNA extraction, each honey sample (500 g total) was homogenized at room temperature by careful manual mixing using a sterile spoon for approximately one minute until complete homogenization to ensure a uniform distribution of pollen grains. For each sample, three technical replicates were taken (labeled A, B, and C). To ensure representative sampling, each technical replicate consisted of two sub-aliquots of 15 g each (e.g., H1 A1 and H1 A2), which were weighed and processed separately during the lysis and extraction steps.

**Figure 1:**
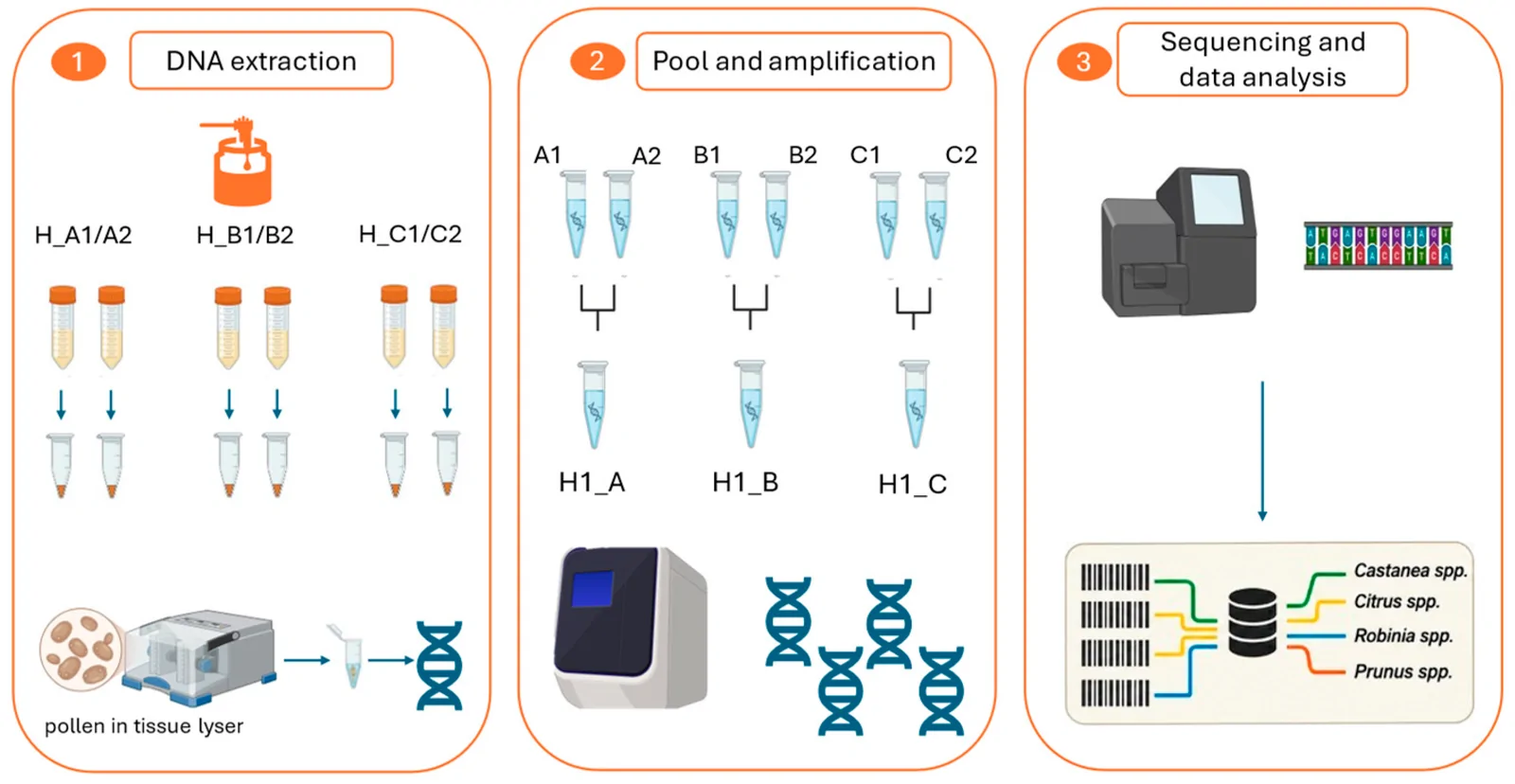
Experimental workflow for each honey sample. The scheme illustrates: (1) sample subdivision into technical duplicates (H_A1/A2, H_B1/B2, and H_C1/C2) and pollen pellet recovery prior to cell lysis via tissue lyser and total DNA extraction; (2) combination and pooling of duplicate DNA extracts into pooled representative samples (H1_A, H1_B, and H1_C) followed by target locus PCR amplification; and (3) Illumina high-throughput sequencing followed by bioinformatic processing, where colored lines denote barcode sequence matching against reference databases for taxonomic botanical assignment (*Castanea* spp., *Citrus* spp., *Robinia* spp., and *Prunus* spp.).

Sample preparation and genomic DNA extraction and purification were performed following the protocol described by Laube et al. [40], integrating an additional post-extraction purification step to optimize template quality. Briefly, each 15 g sub-aliquot was transferred into a 50 mL tube, mixed with 30 mL nuclease-free water (QIAGEN S.r.l., Milan, Italy), and homogenized at 37 °C (500 rpm, 30 min). After centrifugation (10,000 rpm, 30 min), the supernatant was discarded, and the pellet was washed twice by resuspension in 1 mL of nuclease-free water, followed by centrifugation (13,000 rpm, 10 min). The final pellet was resuspended in 180 µL ATL buffer (QIAGEN S.r.l., Milan, Italy) and subjected to mechanical lysis in a TissueLyser II (QIAGEN S.r.l., Milan, Italy) using a 3 mm tungsten bead at 30 Hz for 4 min. DNA was then extracted and purified using the DNeasy Blood & Tissue Kit (QIAGEN S.r.l., Milan, Italy). Resulting DNA from the corresponding paired duplicates was pooled together (Figure 1). Finally, extracted DNA was subject to PCR inhibitor removal (e.g., polyphenolics, pigments, enzymes, flavonoids) using the One-Step PCR Inhibitor Removal Kit (Zymo Research, Freiburg, Germany). DNA quantity and purity were determined spectrophotometrically (A260/280, A260/230; BioPhotometer D30, Eppendorf, Hamburg, Germany). Then, extracted DNA was amplified using primers rbcLa-F and rbcLa-R [41], which amplify a fragment of ~550 bp of the chloroplast *rbcL* gene. Each 20 µL reaction contained 10 µL 2× Phusion High-Fidelity Master Mix (Thermo Fisher Scientific, Milan, Italy), 0.6 µL DMSO, 0.5 µM of each primer, and approximately 20 ng of template DNA. Cycling conditions were as follows: 98 °C for 30 s; 35 cycles of 98 °C for 10 s, 55 °C for 30 s, and 72 °C for 20 s; and a final extension at 72 °C for 7 min. Amplicons were visualized on 1.5% agarose gels stained with Green Gel Safe 10,000× (Thermo Fisher Scientific, Milan, Italy). Extraction (no matrix) and PCR negative controls (no template) were included and evaluated via agarose gel electrophoresis. No visible amplification products were observed in the negative controls. These controls were not subjected to sequencing and were therefore not included in the downstream metabarcoding analysis.

Sequencing of the positive amplicons was performed on an Illumina MiSeq platform (Illumina Inc., San Diego, CA, USA) (2 × 300 bp paired-end) at IGA Technology Services S.R.L. (Udine, Italy).

### 2.5. Bioinformatics Analysis

Paired-end reads were processed using the DADA2 package (version 1.28.0) in R (version 4.3.1) [42]. Raw sequences were denoised and resolved into amplicon sequence variants (ASVs). First, primers were removed, and the remaining reads were used to train the error model using a machine learning approach. Then, forward and reverse reads were dereplicated to generate unique sequences and denoised (collapsed) into amplicon sequence variants (ASVs) by applying the trained error model. Due to the length of the *rbcL* expected amplicon (~550 bp), we prevented the loss of any reads that did not overlap between forward and reverse sequences by applying the justConcatenate = TRUE option during the mergePairs function. After the check for chimeras, the taxonomic assignment was performed using standalone BLAST in the BLAST+ suite (version 2.14.0) [43,44] against a custom *rbcL* reference database. This custom database was constructed and curated using the Creating Reference databases for Amplicon-Based Sequencing (CRABS) software pipeline (version 0.1.0), following the curation criteria previously validated by Ranieri et al. [31]. Briefly, nucleotide sequences were downloaded from NCBI, filtered exclusively for the Streptophyta clade, and strictly curated by removing all entries lacking complete species-level identification (e.g., sequences labeled as “environmental”, “sp.”, “cf.”, or “aff.”).

Assignments with lower than 90% similarity and query coverage lower than 70% of the amplicon length were excluded from the BLAST results. In the BLAST step, for each ASV (query), we generated 3 hits. If the three hits included different species with the same BLAST score, we used the Lowest Common Ancestor (LCA) algorithm [45], and the ASV was assigned to the first common taxonomic rank. In addition, to avoid overestimation of diversity due to biological variations (intra-genomic, intra-individual, intra-species variations), after the taxonomic assignment with BLAST, we applied a post-ASV clustering step. We first ranked the assignments of ASVs into three levels based on similarity with the reference from the BLAST output: (i) assignments showing similarity with the reference sequence in the range 99–100% were considered assigned at the species level; (ii) assignments showing similarity with the reference sequence in the range 95–<99% were considered reliable at the genus level; (iii) assignments showing similarity with the reference sequences lower than 95% were considered reliable at the family level. Finally, the ASVs with the same taxonomic path were collapsed together.

Congruences of taxonomic profiles within the three replicates of each honey sample were explored and their distributions compared using the Kendall and Kruskal–Wallis tests. For the comparisons with label declarations and melissopalynological results, the three replicates were averaged as a single representative taxonomic profile for each sample. To explore community-level variation among technical replicates, beta diversity was evaluated using Bray–Curtis dissimilarity and visualized via Principal Coordinates Analysis (PCoA), accompanied by Permutational Multivariate Analysis of Variance (PERMANOVA). This multivariate analysis was treated as strictly exploratory rather than inferential given the small replicate size. Data processing, visualization, and statistical analyses were performed in R (version 4.3.1) using the packages phyloseq (v1.32) [46], ggplot2 (v3.4.4) [47], and microeco (v 0.19.0) [48]. The scripts used are available as a Supplementary File S1.

Raw sequences were deposited in the Sequence Read Archive (SRA) under BioProject PRJNA1453498.

## 3. Results

### 3.1. Label Analysis

All analyzed samples (*n* = 10) showed 100% compliance with the mandatory information required by Regulation (EU) No. 1169/2011 and D.Lgs. 179/2004, as detailed in Table 2.

### 3.2. Melissopalynological Results

Melissopalynological analysis identified 42 families, 40 genera and 19 species. Furthermore, melissopalynological analysis allowed for the assessment of the botanical origin of the analyzed samples. The declared botanical origin reported on the label was confirmed for six of the ten samples (H1, H5, H6, H7, H9 and H10), while four samples (H2, H3, H4 and H8) required reclassification based on their pollen spectra.

In particular, samples H1 and H8 were classified as unifloral cherry and crimson clover honeys, respectively, due to the predominance of *Prunus* and *Trifolium incarnatum* pollen, which accounted for 72% and 74% of the nectariferous taxa (Table 3). The declared botanical origin of sample H8 as robinia (acacia) honey could not be confirmed [19], as *Robinia pseudoacacia* pollen represented only 1% of the nectariferous taxa, not reaching the 15% pollen content required by the UNI reference method [19] (Table 3). Since no specific melissopalynological reference thresholds are currently available for these honey types, the criterion of a dominant taxon exceeding 45% was applied according to the UNI 11299:2008 [16] standard method.

Sample H2, also declared as cherry honey, showed a pollen spectrum characterized by a high abundance of *Lotus* (63%), while *Prunus* was poorly represented (19%) (Table 3). Although *Lotus* was the dominant pollen type, given its over-represented nature [49], the sample was classified as polyfloral. Sample H4, declared as polyfloral honey, showed a pollen spectrum consistent with that of unifloral coriander honey (Table 3). In particular, the Apiaceae A pollen type, which includes *Coriandrum sativum*, accounted for 67% of the nectariferous pollen spectrum. This value falls within the range reported for coriander honey by Stefano et al. [50].

**Table 3:** Botanical diagnosis and pollen distribution of honey samples. The table compares the declared botanical origin with the melissopalynological diagnosis performed according to UNI reference standards and [6,23]. The column “*Dominant Pollen and Expected Botanical Taxon (%)*” reports, for each sample, the most abundant pollen type identified in the melissopalynological analysis together with its relative percentage, followed, when detected, by the botanical taxon expected according to the declared unifloral origin. Expected taxa are indicated in bold, even when not representing the dominant pollen type. The *Total Pollen Grains (%)* value represents the proportion of nectariferous pollen within the overall pollen spectrum, including over-represented or nectarless taxa. Bold font indicates the botanical taxon expected according to the declared unifloral origin, regardless of whether it represents the dominant pollen type.

Sample	Declared Botanical Origin	Melissopalynological Classification	Dominant Pollen and Expected Botanical Taxon (%)	Total Pollen Grains (%)	Reference
H1	Cherry	Unifloral Cherry	***Prunus*** < 40 µm (72%)	65%	[16]
H2	Cherry	Polyfloral	*Lotus* (63%), ***Prunus*** < 40 µm (19%)	54%	[16]
H3	Polyfloral	Unifloral Chestnut	*Castanea* (90%)	89%	[18]
H4	Polyfloral	Unifloral Coriander	Apiaceae A (67%)	60%	[50]
H5	Chestnut	Unifloral Chestnut	***Castanea*** (98%)	98%	[18]
H6	Chestnut	Unifloral Chestnut	***Castanea*** (98%)	98%	[18]
H7	Robinia (acacia)	Unifloral *Robinia*	*Paliurus* (30%), **Robinia** (17%)	20%	[19]
H8	Robinia (acacia)	Unifloral Crimson Clover	*Trifolium incarnatum* (74%), ***Robinia*** (1%)	69%	[19]
H9	Citrus	Unifloral Citrus	*Prunus* < 40 µm (19%), ***Citrus*** (12%)	9%	[20]
H10	Citrus	Unifloral Citrus	*Lotus* (17%), ***Citrus*** (13%)	8%	[20]

Samples H3, H5 and H6 were classified as chestnut honeys; H7 as robinia (acacia) honey; and H9 and H10 as citrus honeys. Their pollen spectra were compliant with the characterization sheets for Italian and European unifloral honeys [6,23] and with the corresponding UNI standards [18,19,20] (Table 3).

Quantitative analysis supported the botanical diagnosis. Estimated pollen density, expressed as pollen grains per 10 g of honey (GP/10 g), showed considerable variability among samples, ranging from 8300 (H7) to 366,700 (H6). Samples H3, H5 and H6 (chestnut honeys) exhibited the highest pollen densities, whereas H7, H9 and H10 (acacia and citrus honeys) showed markedly lower values, reflecting the intrinsic differences between over-represented and under-represented pollen sources. Detailed quantitative and qualitative data for all samples are provided in Supplementary Table S1. Chestnut honeys were characterized by relatively low pollen diversity and by the frequent occurrence of *Rubus* and *Trifolium alexandrinum* types as accompanying taxa.

Melissopalynological analysis also confirmed the declared Italian geographical origin of all samples. The observed pollen spectra were consistent with their provenance, based on the general pollen composition and on the occurrence of pollen types reported in Italian honeys [35].

### 3.3. Molecular Analyses

Genomic DNA was successfully extracted from all samples (A, B, C). DNA concentrations across samples ranged from 27.4 to 237.5 ng/µL, with robinia (acacia) and polyfloral honeys exhibiting lower concentrations and more variable purity metrics, whereas citrus honeys showed the highest DNA yields. PCR amplification was successful for all samples, except for one H8 replicate (H8 B). The failure of H8 B may have resulted from low DNA yield and/or the presence of PCR-inhibitory compounds; therefore, this replicate was excluded from subsequent analyses. Illumina MiSeq sequencing of the remaining 29 subsamples produced 1,122,202 raw reads. After preprocessing steps and taxonomic assignment, 932,480 reads were retained, corresponding to 605 unique ASVs (Supplementary Table S2), including 1 class, 42 orders, 128 families, and 482 genera, while 42 ASVs reached the species level. Statistical analysis (Kruskal–Wallis test) indicated significant differences in ASV distribution among technical replicates for all samples, except for sample H8 (Supplementary Table S3). However, inspection of the most abundant taxa indicated that the core botanical profile was highly reproducible, with the top five dominant taxa consistently detected across all replicates within each sample (Supplementary Table S4). These findings should nevertheless be interpreted with caution because of the limited number of technical replicates per sample. A complementary exploratory analysis based on Bray–Curtis dissimilarity and PERMANOVA (Supplementary Figure S1) showed that replicate clustering was primarily driven by dominant taxa. However, the absence of statistically significant differences should not be interpreted as evidence of superior replicate consistency. Taken together, these results support the reproducibility of the qualitative core botanical profile across technical replicates.

Overall, the dataset was characterized mainly by entomophile taxa, such as *Castanea* spp. (26.22%), *Prunus* spp. (12.64%), *Papaver rhoeas* (8.12%), *Trifolium alexandrinum* (3.36%), and *Lotus halophilus* (1.78%), alongside high proportions of mainly anemophilous taxa such as *Quercus* spp. (13.86%) and *Pinus* spp. (3%).

**Figure 2:**
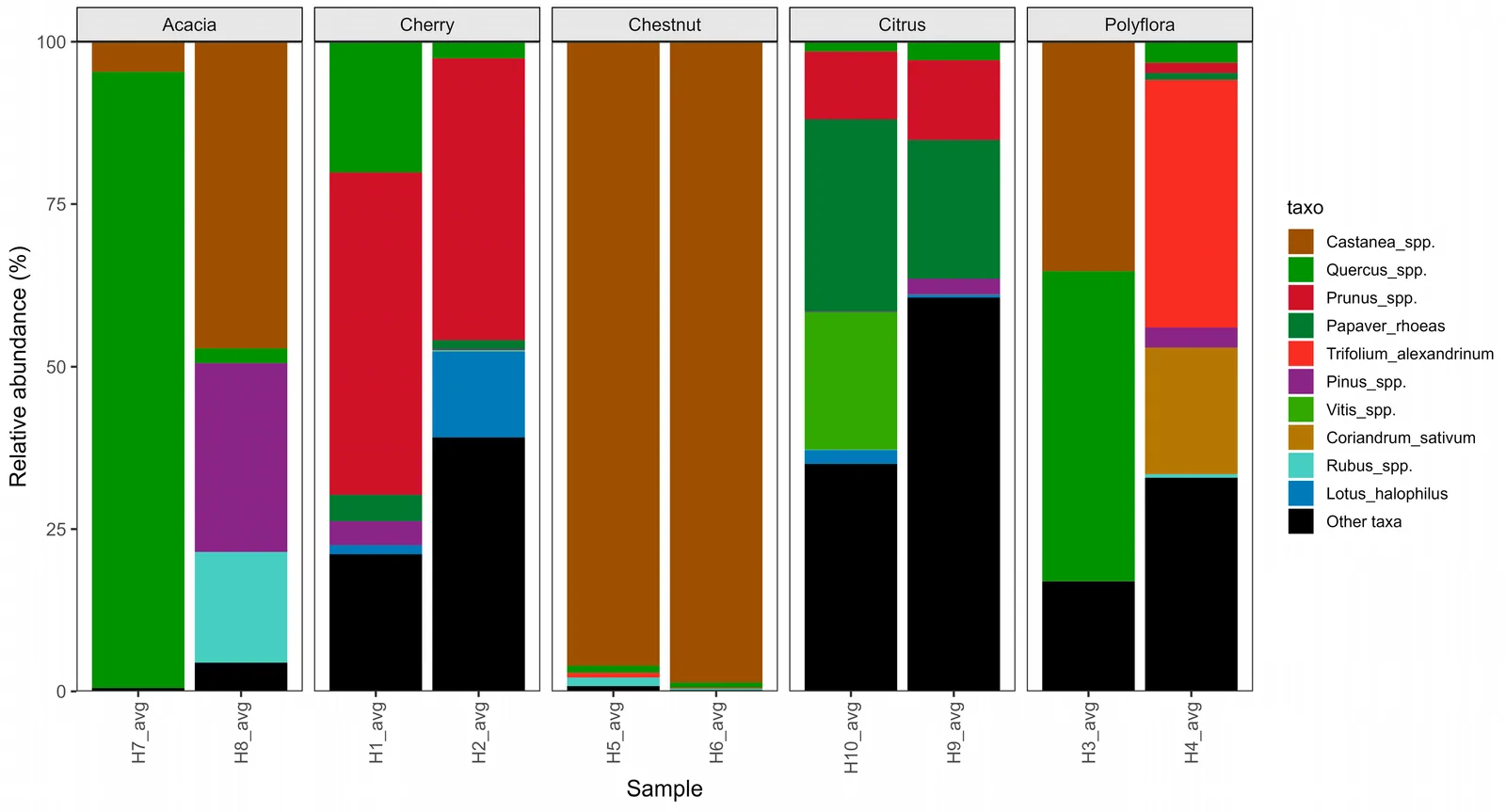
Relative abundance (%) of the ten most abundant taxa across samples, grouped by botanical origin. Remaining taxa were combined into “Other taxa”. Note: In the acacia-labeled samples (H7_avg and H8_avg), *Robinia pseudoacacia* sequences were not detected among the sequence reads.

The botanical profiles of the samples revealed different patterns when compared with their commercial declarations. In cherry (H1 and H2) and chestnut (H5 and H6) honeys, the most abundant taxa were the botanical species corresponding to the commercial name declared on the labels: cherry profiles were characterized by *Prunus* spp. (43–50% of total reads), and chestnut honeys were characterized by a high read count for *Castanea* spp., representing more than 96% of total reads in both samples. In citrus honeys (H9 and H10), the target taxon (*Citrus* spp.) was included in the five most abundant taxa—but with a relative read abundance of ~5% in both samples—which were instead characterized by high read proportions of the non-melliferous species *Papaver rhoeas* (21–29%). The most pronounced discrepancy was observed in Robinia (acacia) honeys (H7 and H8), where *Robinia pseudoacacia* was not detected by metabarcoding. Notably, the most pronounced discrepancy was observed in robinia (acacia) honeys (H7 and H8), where *Robinia pseudoacacia* sequences were undetected in the metabarcoding profiles despite being confirmed or expected by melissopalynological criteria (Table 4 and Figure 2). For the polyfloral honeys (H3 and H4), no specific botanical species was expected based on the commercial declaration on the labels (Table 4 and Figure 2).

### 3.4. Comparison Between Melissopalynology and DNA Metabarcoding

**Figure 3:**
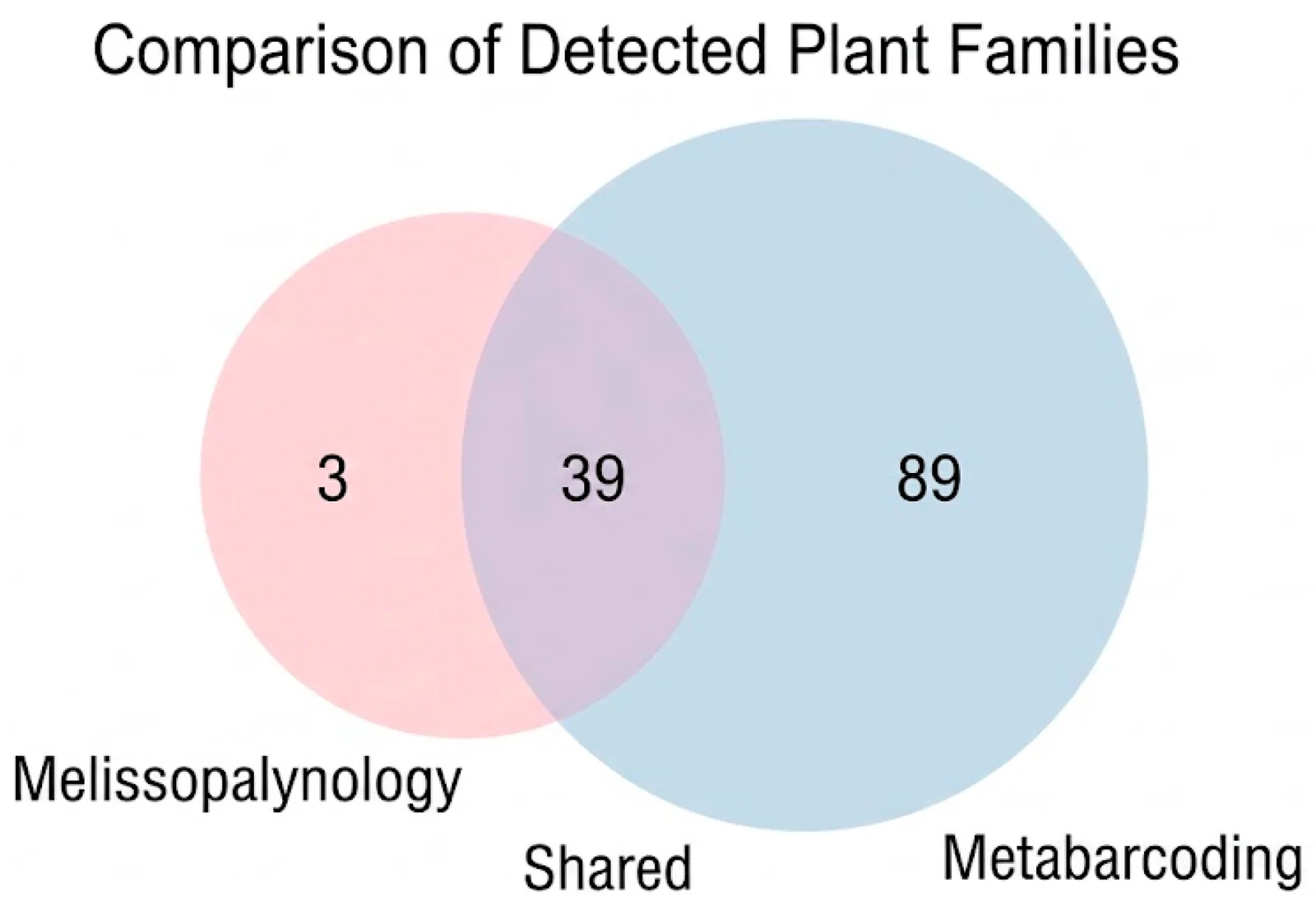
Venn diagram illustrating the overlap and discrepancy in plant family identification between DNA metabarcoding (NGS) and melissopalynological analysis. The central intersection displays the core botanical families detected by both methodologies, while the outer circles list families identified exclusively by each approach. The complete list of these plant families is provided in Supplementary Table S5.

To ensure comparability between melissopalynological and molecular data, the botanical comparison was performed at the family level. Therefore, only taxa that could be reliably assigned to a defined plant family were included in the analysis. Entries classified as *Unidentified* in the melissopalynological analysis, corresponding to pollen grains that could not be assigned to a specific botanical family, were excluded from the overlap analysis. A total of 128 plant families were identified by DNA metabarcoding, whereas 42 families were identified by melissopalynological analysis. Among these, 39 families were detected by both approaches, while 89 families were exclusively detected by metabarcoding and three exclusively by melissopalynology (Figure 3; Supplementary Table S5).

The comparison between declared botanical origin and analytical outcomes obtained through melissopalynological and DNA metabarcoding approaches revealed different levels of agreement between methods. A comparative overview of label compliance assessed by both analytical approaches is summarized in Table 5.

## 4. Discussion

The application of advanced DNA-based approaches, either alone or in combination with traditional methods, has emerged as a powerful tool for ensuring traceability and authenticity across a wide range of food matrices [51,52,53,54]. In this context, our results further confirm the strong potential of these approaches when applied to the honey matrix, particularly in their ability to broaden insights into associated biodiversity [55,56]. At the same time, our findings underline several critical aspects and potential sources of bias, especially regarding the detection and interpretation of quantitative botanical profiles [57].

### 4.1. Comparative Analysis of DNA Metabarcoding and Melissopalynology

A direct comparison between melissopalynology and metabarcoding required aligning the taxonomic resolution of the two approaches. This is not trivial since melissopalynology often provides identifications at the family level, while metabarcoding frequently achieves genus- or species-level assignments. Therefore, metabarcoding results were aggregated to the corresponding family rank, enabling a consistent evaluation of concordance between methods. Overall, 39 families were consistently detected by both methods, confirming that diagnostically relevant taxa are reliably captured [11]. Notably, these shared families include the principal nectariferous and entomophilous taxa dominating the Apulian landscape, accounting for a large fraction of the regional taxonomic diversity. This overlap suggests that, despite methodological differences, both approaches converge on the core botanical structure of the honey matrix. However, considerable numbers of families were detected exclusively via DNA metabarcoding (*n* = 89) (Figure 3). The detection of these additional taxa highlights the sensitivity of the molecular approach, as it captures under-represented biodiversity that often escapes microscopic sedimentation of melissopalinology. These include anemophilous taxa (e.g., Ericaceae, Juglandaceae, and Moraceae) and families with low pollen production (e.g., Orchidaceae and Cucurbitaceae) [56,57], as observed in our dataset (Supplementary Table S5). Notably, the exclusive detection of families such as Capparaceae, which is typical of the Mediterranean maquis, and Cannabaceae, which is linked to local agricultural landscapes, provides a more detailed geographical profile of Apulia [35,58,59]. Nevertheless, this broader taxonomic recovery must be interpreted cautiously, as it may be influenced by the unfiltered capture of background environmental DNA (e-DNA), rather than actual nectar contribution [60]. Conversely, families identified only through melissopalynology (e.g., Amaryllidaceae, Anacardiaceae, and Cornaceae) confirmed potential biases related to the metabarcoding approach, such as lack of primer universality and incompleteness of reference databases [60,61,62].

The comparison between commercial declarations and analytical outcomes revealed patterns of agreement with relevant regulatory and economic implications (Table 5). Melissopalynological evaluation resulted in the reassignment of several samples according to UNI criteria and to Persano Oddo [6,23] characterization sheets; however, it is essential to distinguish true labeling non-compliance from conservative commercial designation. In some cases, honeys marketed as polyfloral fulfilled the analytical requirements for unifloral classification. Such situations do not represent mislabeling, as the declaration of polyfloral origin remains fully compliant in the absence of analytical verification. Rather, these findings highlight a potential missed opportunity for producers, since analytical characterization may enable the recognition of unifloral honeys, offering potential for higher commercial value and improved market differentiation. This aspect is exemplified by samples H3 and H4, both commercially labeled as polyfloral but consistently identified as unifloral honeys by melissopalynological analysis and DNA metabarcoding, corresponding respectively to chestnut and coriander honey. Crucially, marketing a honey that technically meets unifloral criteria under the broader designation of “polyfloral” does not constitute regulatory mislabeling or fraud. However, considering that these unifloral typologies generally achieve higher market value compared with polyfloral products [4,5,6,7], these cases illustrate how analytical verification may support product valorization and economic differentiation rather than merely serving as a regulatory control tool. The adoption of routine botanical profiling could therefore represent a valuable strategy for beekeepers, enabling accurate product characterization and ensuring fair market practices without implying fraudulent intentions.

From a legislative standpoint, these two approaches revealed discrepancies. Melissopalynology revealed labeling non-compliances for unifloral samples H2 and H8, but only one (H8) was confirmed as non-compliant by the metabarcoding approach. Although such discrepancies do not necessarily imply intentional fraud, they may affect market transparency and consumer confidence, particularly in higher-end honey categories where botanical origin directly determines product value. In this context, the integration of complementary analytical approaches can strengthen botanical diagnosis, particularly when analytical methods provide divergent results. These observations highlight the importance of analytical verification in ensuring consistency between commercial designation and measurable botanical composition, thereby supporting fair market practices and consumer protection.

Our metabarcoding results further highlight that methodological discordances should be interpreted cautiously. The absence of expected taxa in molecular profiles (e.g., H7 and H8), despite confirmation by melissopalynology, likely reflects biological and technical constraints associated with pollen under-representation, such as *Robinia pseudoacacia*, as well as differential DNA recovery efficiency or marker-related limitations rather than actual botanical absence. This aspect is particularly relevant for nectar sources characterized by low pollen production, where molecular detectability may be reduced despite compliance with palynological criteria. Overall, the combined interpretation of morphological and molecular evidence strengthens the reliability of botanical attribution, while illustrating how complementary analytical approaches can simultaneously support regulatory assessment and economic valorization of apicultural products.

### 4.2. Geographical Traceability and Landscape Signatures

The botanical profiling produced by melissopalynology and the molecular approach provided evidence consistent with the declared Italian geographical origin. Indeed, the detected taxa reflect botanical elements commonly associated with European honey profiles, as described in classical melissopalynological studies [6] and confirmed by recent works integrating pollen data with chemical analyses [63,64]. Specifically, the detection of *Papaver* spp., *Trifolium* spp., *Melilotus* spp., *Vicia* spp., *Astragalus* spp., *Medicago* spp., and *Lotus* spp. in most sample-specific patterns is highly consistent with the herbaceous and agricultural matrix characteristic of Mediterranean environments. Furthermore, the consistent and abundant representation of *Quercus* spp. across most samples aligns well with the airborne pollen background and the semi-natural habitats typical of the Apulia region [58,59], highlighting the high sensitivity of the molecular approach in capturing the regional environmental DNA signal. These compositional patterns also reflect nomadic beekeeping practices. The periodic relocation of hives to follow flowering cycles exposes colonies to multiple floral landscapes, generating complex molecular signatures [65]. In our study, this is particularly evident in samples H6 and H7, where the molecular and melissopalynological profiles revealed a transition from strictly coastal Mediterranean flora to taxa more typical of internal, hilly environments, such as a significant presence of *Trifolium alexandrinum* and *Castanea* spp. We hypothesize that the detection of these species, alongside the airborne pollen background, suggests that nomadic practices expanded the foraging range toward neighboring ecological corridors, creating a composite botanical record. However, since there is no precise data on the location of the apiaries, and it is not possible to accurately map where the bees go to forage, this dynamic remains a hypothesis. This mobility generates a composite ecological record, a synthesis of multiple nectar sources, which makes the establishment of rigid “reference” profiles for regulatory purposes inherently challenging [11,65,66]. Ultimately, although the small sample size (*n* = 10) does not allow us to rule out with certainty the presence of commercial honey blends, the high botanical diversity observed in this dataset, especially via metabarcoding, revealed no traces of pollen from outside the country, as also confirmed by melissopalinological data; on the contrary, it accurately reflects the high biodiversity that characterizes Mediterranean ecosystems.

In the context of international markets, such geographically informative botanical signatures may also have relevant applications for honey authentication and the protection of high-value products. DNA metabarcoding may provide a complementary tool for supporting geographical-origin verification by revealing rare, under-represented, or regionally characteristic plant taxa associated with specific production areas [28,56]. The combined occurrence of multiple geographically informative taxa could contribute to the definition of region-specific molecular signatures useful for assessing the consistency of a honey sample with its declared geographical origin [2,28]. Following validation on larger and geographically representative datasets, integration of these DNA-based signatures with established analytical approaches could support the protection and traceability of PDO honeys in international markets [2,25].

Since geographical traceability relies on the preservation of authentic botanical signatures, the detection of plant taxa unrelated to the production area may also provide indirect evidence of fraudulent practices. Separately, DNA-based botanical profiles may also provide indirect information relevant to syrup-based adulteration. Notably, neither melissopalynological analysis nor DNA metabarcoding revealed botanical signatures attributable to common exogenous sugar-syrup source crops, such as rice (*Oryza sativa*), maize (*Zea mays*), or sugar beet (*Beta vulgaris*). Such syrups are increasingly reported within the honey supply chain as a source of adulteration, either through direct addition for economic gain [67] or indirectly via supplemental bee feeding during nectar flow to artificially increase honey yield [68,69,70]. While the absence of these markers suggests that none of the investigated samples contained detectable traces of these common adulterants, this result must be interpreted with caution. Highly processed syrups often contain severely degraded DNA; therefore, negative molecular outcomes do not unequivocally guarantee the absence of adulteration. The present results should therefore be interpreted only as the absence of detectable DNA signatures from these specific plant sources, rather than as evidence that syrup adulteration was excluded.

### 4.3. Challenges in Traceability of Honey Matrices

Despite the promising applications highlighted above, the interpretation of botanical and geographical signatures in honey remains intrinsically complex due to biological and methodological constraints associated with the honey matrix itself. Botanical profiling of honey matrices is not easy since a direct proportion between the nectar contribution of a plant to the composition of honey and the quantity of its pollen in the honey matrix is not possible. To mitigate biases due to over- and under-represented pollens, official UNI standards used in melissopalynology define quantitative thresholds of characteristic pollen types only for certain unifloral honeys (e.g., ≥90% *Castanea sativa* for chestnut honey, ≥15% *Robinia pseudoacacia* for robinia (acacia) honey, and ≥10% *Citrus* spp. for citrus honey). For other types, such as *Prunus* spp. (cherry honey), *Tilia* spp. (linden honey), *Thymus* spp. (thyme honey), and *Lavandula* spp. (lavender honey), indicative ranges have been proposed (≥25% *Prunus*, ≥20–30% *Tilia*, and ≥15–30% *Lavandula*) and are widely adopted by reference laboratories [8,35]. In addition, melissopalynology applies technical filtering during the analyses, and anemophilous and nectarless taxa (e.g., *Quercus* spp., *Pinus* spp., *Olea* spp.) are used only to evaluate the geographical origin, but they are typically excluded from the relative frequency calculation used to define the botanical origin [16].

However, the metabarcoding approach captures the entire genetic signature of the matrix regardless of the pollen’s nectariferous role [26], meaning that morphological mitigation strategies for over- and under-represented pollens cannot be applied. In addition, methodological biases may arise at several steps (e.g., honey matrix sampling, DNA extraction efficiency, and amplification processes), which can affect both taxa detection and the relative abundance of reads obtained [2,71]. For this reason, DNA metabarcoding should be considered a semi-quantitative tool rather than a fully quantitative one. In addition, the number of sequence reads does not directly reflect the original proportion of pollen grains in the sample, as it is influenced by differential DNA recovery, marker copy number, primer affinity, and amplification efficiency. These limitations in DNA-based quantification have been widely documented across different food matrices [72,73,74] and must be carefully considered when interpreting botanical profiles derived from metabarcoding data [2,71]. Furthermore, the intrinsic heterogeneity in the viscosity of the honey matrix leads to an uneven spatial distribution of pollen grains, causing quantitative variability among extraction aliquots [11]. These stochastic sampling effects underscore a well-known limitation of e-DNA metabarcoding in complex matrices: the combination of low-biomass traces, differential extraction efficiencies, and PCR amplification biases heavily distorts the detection of rare taxa. Consequently, while the core dominant components remain comparatively stable and reproducible across technical replicates, the “long tail” of rare environmental DNA exhibits significant random fluctuations. For biodiversity assessment, rare taxa should therefore be considered with particular caution, as this fraction may include methodological artifacts and ornamental or incidental taxa, as well as genuinely low-abundance environmental signals that are easily masked by hyper-represented species such as *Castanea* and *Quercus*, as observed in the present study. In this regard, a further methodological limitation is that extraction blanks and PCR-negative controls, although negative by gel electrophoresis, were not sequenced. Thus, low-level background contamination cannot be completely excluded, particularly for rare sequence variants, although the absence of visible amplification argues against substantial contamination during DNA extraction and PCR amplification. These findings are consistent with previous reports showing that both traditional and molecular approaches can be affected by stochastic sampling limitations, leading to reduced reliability in the detection of low-abundance taxa [11,75].

In honey matrices, this phenomenon is particularly critical for the detection of nectar sources that are pollen-poor. This may explain the lack of detection observed in the two robinia (acacia) honey samples, in which *Robinia pseudoacacia* was not identified even at the genus-level profile but was dominated by *Quercus* spp., which accounted for 95% of the total reads. The high proportion of anemophilous taxa (*Quercus* spp., in this case) likely reflects the high environmental pressure of airborne pollen and cannot be avoided by metabarcoding, and it could obscure the presence of rarer taxa. Crucially, this lack of detection cannot be attributed to a technical failure or primer mismatch. The employed primer pairs have been previously evaluated in silico by Ranieri et al. [31], and their amplification capability with respect to *R. pseudoacacia* was empirically confirmed in the present study by the successful detection of *Robinia* traces in sample H4. The failure to detect *Robinia pseudoacacia* may also be linked to biological factors affecting DNA availability in pollen grains. Plastid DNA is known to undergo active degradation during pollen maturation through the action of the exonuclease DPD1, a mechanism conserved among seed plants [76]. Because plastid-based metabarcoding relies on the presence of intact chloroplast DNA, this degradation can substantially reduce the amount of amplifiable template, particularly in pollen-poor nectar sources, thereby further limiting their detectability in complex matrices such as honey [77]. Consistent with this hypothesis, several metabarcoding studies have reported difficulties in detecting *R. pseudoacacia*, regardless of the genetic marker employed [2,65,78].

Interestingly, the detection of *Citrus* spp., another economically relevant honey but under-represented pollen type, was achieved in both samples, although at low relative percentages (~5%) (Table 4). This observation supports the notion that, within a metabarcoding workflow, biases related to pollen over- or under-representation may interact with additional pollen grain characteristics. Indeed, differences in pollen grain morphology and exine architecture can strongly influence DNA extraction efficiency, potentially distorting botanical profiles in complex and unpredictable ways [29,79,80,81,82]. By contrast, in cherry and chestnut honeys, the consistent detection of *Prunus* spp. and *Castanea* spp. was associated with their dominance in terms of read abundance. Therefore, in the case of unifloral honeys derived from taxa with normally or over-represented pollen, metabarcoding results may reasonably reflect the actual pollen composition of the samples [11,60].

In addition, metabarcoding outcomes can be influenced by the complex chemical nature of the honey matrix. Despite the application of dedicated purification and clean-up steps during DNA extraction to minimize interference, the diverse array of secondary metabolites, including organic acids, polyphenols, pigments, enzymes, flavonoids, and solid particles such as waxes, may still exert a residual inhibitory effect. These compounds are known to interact with DNA polymerases, potentially leading to taxa-specific amplification biases even when overall PCR success is achieved [2,32].

Thus, while melissopalynological analysis remains the standard for commercial classification, molecular profiling provides a broader complementary biodiversity profile [32]. By detecting a broader range of environmental DNA, including rare or low pollen-producing taxa that may be under-represented in microscopic sediment [83], metabarcoding may serve as a highly sensitive complementary tool for geographical profiling, offering a more detailed map of the surrounding landscape [28].

### 4.4. Marker Choice and Taxonomic Resolution

The selection of the *rbcL* marker allowed for the detection of 128 plant families. However, our results also confirmed the well-known resolution limits of this plastid region: species-level discrimination was unambiguously possible for only 42 ASVs. This high sequence conservation, while highly beneficial for universal primer binding across diverse land plants, inherently limits the ability to distinguish closely related species within complex botanical genera [33,34].

The use of *rbcL* also entails some limitations in terms of species-level discrimination, as the relatively conserved nature of this region may prevent the unambiguous differentiation of closely related taxa. More variable markers, such as ITS2, may provide higher taxonomic resolution, while *trnL* has also been successfully applied in plant and pollen metabarcoding. However, each marker presents specific advantages and limitations in terms of taxonomic coverage, amplification performance and discriminatory power. Therefore, the use of *rbcL* in the present preliminary study was intended to provide a broad and consistent botanical profile of the investigated honey samples, while the integration of complementary markers may represent a valuable approach for future studies requiring enhanced species-level resolution.

In line with our recent in silico evaluations [31] and other similar studies [84,85], the use of the *rbcL* marker alone represents a robust tool for baseline botanical profiling. Nevertheless, we recommend that future studies integrate *rbcL* profiles with more variable nuclear regions, such as ITS2, or additional markers, such as trnL, when absolute species-level precision is required for definitive geographical traceability. Integrating molecular profiles with melissopalynology and geographical information remains essential to mitigating technical biases and to supporting the realistic application of DNA tools for robust honey characterization.

## 5. Conclusions

In this preliminary study, the botanical and geographical profiles of honey were assessed through the integration of traditional melissopalynology and DNA metabarcoding. The results show that, although each method presents inherent limitations, their combined application constitutes a robust and complementary strategy for honey characterization. Conventional melissopalynological analysis remains the gold standard for botanical and geographical origin assessment and a cornerstone of routine honey testing due to its scalability, cost-effectiveness, and regulatory acceptance. However, DNA metabarcoding significantly expands the detectable biodiversity, providing a broader environmental DNA footprint of plant species associated with specific geographic production areas. Notably, the ability of metabarcoding to detect rare or unexpected taxa may contribute to a more refined assessment of geographical origin and could support, in future studies, the development of complementary approaches for authenticity assessment and risk-based control strategies. Potential applications may include the investigation of associations between botanical profiles; foraging landscapes; and exposure to environmental contaminants, pesticide residues, or veterinary drugs. However, these aspects require dedicated experimental validation.

Furthermore, while the detection of highly processed sugar syrups remains challenging due to severe DNA degradation, molecular tools may provide indirect indications through the detection of botanical markers associated with syrup-source crops or taxa inconsistent with the declared geographical origin. Such evidence should therefore be interpreted cautiously and cannot be considered definitive proof of adulteration.

As highlighted by this pilot study, several methodological aspects require further investigation, including the semi-quantitative nature of metabarcoding, stochastic sampling effects, PCR inhibition and marker-related biases. Future studies should therefore include larger and geographically representative sampling, together with multi-marker metabarcoding and appropriate mock-community and artificial honey spike-in controls to assess recovery and matrix-related PCR inhibition.

Overall, these preliminary findings support the potential value of DNA metabarcoding as a complementary tool to melissopalynology. Further validation through larger-scale and standardized studies will be required before its possible integration into official control strategies. From this perspective, the future integration of validated DNA-based methodologies with melissopalynology could strengthen honey authenticity verification, support competent authorities in the development of risk-based food control strategies, and contribute to the valorization and traceability of local products in international markets.

## Figures and Tables

**Table 1:** Information provided on the label of honey subject to sampling.

Sample	Commercial Designation	Declared Geographical Origin
H1	Cherry honey	Italy
H2	Cherry honey	Apulia
H3	Polyfloral honey	Italy
H4	Polyfloral honey	Apulia
H5	Chestnut honey	Italy
H6	Chestnut honey	Apulia
H7	Robinia (acacia) honey	Italy
H8	Robinia (acacia) honey	Italy
H9	Citrus honey	Italy
H10	Citrus honey	Apulia

**Table 2:** Compliance of honey samples with EU and National labeling requirements.

Mandatory Information	Regulatory Reference	Compliance (*n* = 10)	Notes
Product name (e.g., honey)	Dir. 2001/110/EC; D.Lgs. 179/2004	100% (10/10)	Correct denomination used
Botanical/geographical origin	Dir. 2001/110/EC; D.Lgs. 179/2004	100% (10/10)	Voluntary for botanical; mandatory for country
Name/address of operator	Reg. (EU) No. 1169/2011	100% (10/10)	Producer or packager clearly identified
Net quantity	Reg. (EU) No. 1169/2011	100% (10/10)	Expressed in grams or kilograms
Minimum durability date	Reg. (EU) No. 1169/2011	100% (10/10)	“Best before…” format followed
Lot number/batch code	Dir. 2011/91/EU; D.Lgs. 231/2017	100% (10/10)	For traceability purposes
Country of origin	Reg. (EU) No. 1169/2011	100% (10/10)	“Italy” or “Apulia (Italy)”

**Table 4:** Summary of honey profiles of samples, including the commercial denomination, declared botanical source, and molecular identification based on DNA metabarcoding. For each sample, up to five of the most abundant taxa are shown as a percentage. The Scientific Name column refers to the plant species of interest according to the corresponding UNI standard for unifloral honeys. The expected plant species is shown in bold.

Sample ID	Commercial Designation	Expected Botanical Species (Scientific Name)	Molecular Identification
H1 avg	Cherry honey	*Prunus* spp.	***Prunus* spp. 50%**, *Quercus* spp. 20.1%, *Parietaria* spp. 6%, *Papaver rhoeas* 4%, *Pinus* spp. 3.7%
H2 avg	Cherry honey	*Prunus* spp.	***Prunus* spp. 43%**, *Lotus halophilus* 13%, *Lotus* spp. 8%, *Osyris* spp. 7%, *Osyris alba* 4.6%
H3 avg	Polyfloral honey	N/A	*Quercus* spp. 47.8%, *Castanea* spp. 35%, *Convolvulus arvensis* 13.4%, *Convolvulus* spp. 3.5%,
H4 avg	Polyfloral honey	N/A	*Trifolium alexandrinum* 38%, *Coriandrum sativum* 19.5%, *Paliurus* spp. 8.5%, *Eucalyptus* spp. 8.4%, *Tamarix* spp. 4.7%
H5 avg	Chestnut honey	*Castanea sativa*	***Castanea* spp. 96%**, *Rubus* spp. 1.3%, *Quercus* spp. 1%, *Trifolium alexandrinum* 0.7%, *Trifolium* spp. 0.3%
H6 avg	Chestnut honey	*Castanea sativa*	***Castanea* spp. 98%**, *Quercus* spp. 0.8%, *Rubus* spp. 0.2%, *Trifolium alexandrinum* 0.1%
H7 avg	Robinia (acacia) honey	*Robinia pseudoacacia*	*Quercus* spp. 95%, *Castanea* spp. 4.6%, *Gleditsia* spp. 0.4%
H8 avg	Robinia (acacia) honey	*Robinia pseudoacacia*	*Castanea* spp. 47%, *Pinus* spp. 29%, *Rubus* spp. 17%, *Trifolium incarnatum* 4.3%, *Quercus* spp. 2.3%
H9 avg	Citrus honey	*Citrus* spp.	*Papaver rhoeas* 21.3%, *Melilotus* spp. 13.5%, *Prunus* spp. 13%, *Cistus* spp. 6%, ***Citrus* spp. 5%**
H10 avg	Citrus honey	*Citrus* spp.	*Papaver rhoeas* 30%, *Vitis* spp. 21%, *Prunus* spp. 10.5%, ***Citrus* spp. 5.6%**, *Papaver* spp. 4%

**Table 5:** Comparative assessment of label compliance based on melissopalynological and DNA metabarcoding analyses. Label compliance was evaluated according to UNI standards for melissopalynology and qualitatively assessed for metabarcoding based on the detection of the taxon associated with the declared botanical origin (note that sequence detection confirms presence, but does not equate to unifloral compliance). N/A indicates samples marketed as polyfloral honeys, for which unifloral compliance criteria are not applicable.

Sample ID	Declared Botanical Origin	Expected Botanical Taxon	Melissopalynological Classification	Label Compliance (Melissopalynology)	Target Taxon Detected (Metabarcoding)	Target Taxon Presence (Metabarcoding)
H1	Cherry honey	*Prunus* spp.	Cherry honey	YES	*Prunus* spp.	YES
H2	Cherry honey	*Prunus* spp.	Polyfloral honey	NO	*Prunus* spp.	YES
H3	Polyfloral honey	N/A	Chestnut honey	N/A	*Castanea* spp.	N/A
H4	Polyfloral honey	N/A	Coriander honey	N/A	*Trifolium alexandrinum*	N/A
H5	Chestnut honey	*Castanea* spp.	Chestnut honey	YES	*Castanea* spp.	YES
H6	Chestnut honey	*Castanea* spp.	Chestnut honey	YES	*Castanea* spp.	YES
H7	Robinia (acacia) honey	*Robinia pseudoacacia*	Robinia (acacia) honey	YES	NO	NO
H8	Robinia (acacia) honey	*Robinia pseudoacacia*	Crimsom Clover honey	NO	NO	NO
H9	Citrus honey	*Citrus* spp.	Citrus honey	YES	*Citrus* spp.	YES
H10	Citrus honey	*Citrus* spp.	Citrus honey	YES	*Citrus* spp.	YES

## Data Availability

The original contributions presented in this study are included in the article. Further inquiries can be directed to the corresponding authors.

## Supplementary Materials

The following supporting information can be downloaded at https://www.mdpi.com/article/10.3390/foods15193542/s1: Supplementary File S1: Honey metabarcoding analysis pipeline. Table S1: Comprehensive melissopalynological profiles and quantitative parameters of honey samples. The table provides the complete pollen spectrum and quantitative data for each sample. Values for individual pollen taxa are expressed as a percentage of the total nectariferous pollen count. Table S2: Detailed pollen spectrum and relative frequencies of botanical taxa identified in honey samples with melissopalinology analysis. Table S3: Kendall and Kruskal–Wallis tests assessing reproducibility among technical replicates (A, B, C) for each honey sample. Table S4: Top five most abundant taxa identified in each honey sample included in the statistical reproducibility analysis. Table S5: List of families exclusive to melissopalynology and exclusive to NGS, and list of families shared between the two approaches. Supplementary Figure S1: Exploratory Principal Coordinates Analysis (PCoA) based on Bray–Curtis dissimilarity among technical replicates. Supplementary File S2: Representative agarose gel electrophoresis showing PCR amplicon generation (~550 bp) and negative controls. GEL 1: Lane 1, GeneRuler 100 bp DNA Ladder (Thermo Scientific™, Waltham, MA, USA); lanes 2–20, positive samples. GEL 2: Lane 1, GeneRuler 100 bp DNA Ladder (Thermo Scientific™, Waltham, MA, USA); lanes 2–4, positive samples; lane 5, negative sample; lanes 6–12, positive samples; lanes 13–14, negative extraction controls; lane 15, PCR negative control.
