# Supplementary material for: Assessing Botanical Authenticity of Honey Through DNA-Metabarcoding-Based Pollen Analysis: A Preliminary Study
Source: Foods. 2026 Oct 4;15(19):3542. doi: 10.3390/foods15193542 (PMC13664935; doi:10.3390/foods15193542)
Supplement: Supplementary file 1 [file foods-15-03542-s001.zip › Supplementary File S1.pdf]

```r
#Honey Metabarcoding Analysis Pipeline

#Package Installation

if (!requireNamespace("BiocManager", quietly = TRUE)) {
  install.packages("BiocManager")
}

BiocManager::install("dada2")


#Load Libraries

library(dada2)
library(phyloseq)
library(diverse)
library(ggplot2)
library(scales)
library(dplyr)
library(ggpubr)
library(grid)
library(gridExtra)
library(cowplot)
library(microeco)
library(file2meco)
library(tidyverse)
library(eulerr)
library(RColorBrewer)
library(tibble)
```

```
#DADA2 Processing

path <- "D:/analisi_dati_miele/data"
derepRs <- derepFastq(filtRs, verbose = TRUE)

names(derepFs) <- sample.names
names(derepRs) <- sample.names

# Denoising
dadaFs <- dada(derepFs, err = errF, multithread = FALSE)
dadaRs <- dada(derepRs, err = errR, multithread = FALSE)

# Merge paired reads
mergers <- mergePairs(
  dadaFs,
  derepFs,
  dadaRs,
  derepRs,
  justConcatenate = TRUE,
  verbose = TRUE
)

# Sequence table
seqtab <- makeSequenceTable(mergers)

# Length filtering
seqtab <- seqtab[, nchar(colnames(seqtab)) %in% seq(254, 451)]
```

```
# Chimera removal
seqtab.nochim <- removeBimeraDenovo(
  seqtab,
  method = "consensus",
  multithread = FALSE,
  verbose = TRUE
)

# Read tracking
getN <- function(x) sum(getUniques(x))

track <- cbind(
  out,
  sapply(dadaFs, getN),
  sapply(dadaRs, getN),
  sapply(mergers, getN),
  rowSums(seqtab.nochim)
)

colnames(track) <- c(
  "input",
  "filtered",
  "denoisedF",
  "denoisedR",
  "merged",
  "nonchim"
)

rownames(track) <- sample.names
```

```
write.table(track,
        file = "tab_reads_280_250.txt",
        sep = "\t",
        quote = FALSE)

# Export ASV table
seqtab.nochimT <- t(seqtab.nochim)

write.table(seqtab.nochimT,
        file = "ASV_table_honey_280_250.txt",
        sep = "\t",
        quote = FALSE)



# Downstream Analysis


otu_mat <- read.table(
TAX_impa <- tax_table(tax_mat)
sample <- sample_data(sample_df)

lucia <- phyloseq(OTU_impa, TAX_impa, sample)

# Family-level agglomeration
phy_fam <- tax_glom(lucia, "Family")

# Convert to microeco format
meco_lucia_fam <- phyloseq2meco(phy_fam)
```

```
# Export agglomerated OTU table
ps_glom <- tax_glom(phy_fam, taxrank = "Family")

otu_tab <- as.data.frame(otu_table(ps_glom))
tax_tab <- as.data.frame(tax_table(ps_glom))

otu_tax_df <- cbind(tax_tab, otu_tab)

write.table(
  otu_tax_df,
  file = "otu_table_family_glom.txt",
  row.names = TRUE
)

# Relative abundance
impa_perc_cento <- transform_sample_counts(
  phy_fam,
  function(x) x / sum(x) * 100
)

phyloseq_biom3 <- phyloseq::otu_table(impa_perc_cento) %>%
  as.data.frame() %>%
  rownames_to_column("id") %>%
  left_join(
    phyloseq::tax_table(impa_perc_cento) %>%
      as.data.frame() %>%
      rownames_to_column("id")
  )
```

```
write.table(
  phyloseq_biom3,
  file = "phyloseq_fam_xcento_honeybee.txt",
  sep = "\t",
  quote = FALSE
)

# Alpha diversity
summary_alpha <- estimate_richness(
  lucia,
  measures = c("Observed")
)

write.table(
  summary_alpha,
  file = "Summary_alpha.txt",
  sep = "\t",
  quote = FALSE
)

# Stacked Barplot

# Transform to relative abundance

legend_order <- c(setdiff(legend_order, "Others"), "Others")

plot_data$Family_plot <- factor(
  plot_data$Family_plot,
  levels = legend_order
```

```
)

# Color palette
n_taxa <- length(levels(plot_data$Family_plot))

pal <- c(
  "#8C510A", "#01665E", "#5AB4AC", "#C51B7D",
  "#4D9221", "#8073AC", "#E66101", "#DFC27D",
  "#80CDC1", "#B2182B", "#000000"
)

if (n_taxa > length(pal)) {
  pal <- colorRampPalette(brewer.pal(8, "Set2"))(n_taxa)
}

plot_colors <- pal[seq_len(n_taxa)]
names(plot_colors) <- levels(plot_data$Family_plot)

if ("Others" %in% names(plot_colors)) {
  plot_colors["Others"] <- "#000000"
}

# Plot
p <- ggplot(
  plot_data,
  aes(x = Sample, y = Abundance, fill = Family_plot)
) +
  geom_bar(stat = "identity", width = 0.9) +
  facet_grid(
```

```
    . ~ Botanical_origin,
    scales = "free_x",
    space = "free_x"
  ) +
  scale_fill_manual(values = plot_colors, name = "Family") +
  labs(x = "Sample", y = "Relative abundance (%)") +
  scale_y_continuous(
    limits = c(0, 100),
    expand = c(0, 0)
  ) +
  theme_bw(base_size = 14) +
  theme(
    strip.background = element_rect(
      fill = "grey90",
      color = "black"
    ),
    strip.text = element_text(size = 12),
    panel.grid = element_blank(),
    axis.text.x = element_text(
      angle = 90,
      hjust = 1,
      vjust = 0.5
    ),
    legend.position = "right"
  )

print(p)

# Save figures
```

```r
ggsave(
  "stacked_barplot_top10_GLOBAL.png",
  p,
  width = 13,
  height = 7,
  dpi = 600
)

ggsave(
  "stacked_barplot_top10_GLOBAL.pdf",
  p,
  width = 13,
  height = 7
)

#Venn Diagram

venn_counts <- c(
  "NGS" = 91,
  "Melissopalynology" = 7,
  "NGS&Melissopalynology" = 37
)

venn_plot <- plot(
  euler(venn_counts),
  quantities = list(cex = 1.2, font = 1),
  labels = list(cex = 1.1, font = 2),
  fills = list(
    fill = c("#C2E2D4", "#F5D6C6"),
```

```
    alpha = 0.8
  ),
  edges = list(col = "#7F9B8D", lwd = 1)
)

print(venn_plot)

tiff(
  "Figure_3_Venn.tiff",
  width = 8,
  height = 6,
  units = "in",
  res = 300,
  compression = "lzw"
)

print(venn_plot)
dev.off()
```
