# Supplementary material for: Assessing Botanical Authenticity of Honey Through DNA-Metabarcoding-Based Pollen Analysis: A Preliminary Study
Source: Foods. 2026 Oct 4;15(19):3542. doi: 10.3390/foods15193542 (PMC13664935; doi:10.3390/foods15193542)

Supplementary File S2: Representative agarose gel electrophoresis showing PCR amplicon generation (~550 bp) and negative controls. **GEL 1:** Lane 1, GeneRuler 100 bp DNA Ladder (Thermo Scientific™, USA); lanes 2–20, positive samples. **GEL 2:** Lane 1, GeneRuler 100 bp DNA Ladder (Thermo Scientific™, USA); lanes 2–4, positive samples; lane 5, negative sample; lanes 6–12, positive samples; lanes 13–14, negative extraction controls; lane 15, PCR negative control.

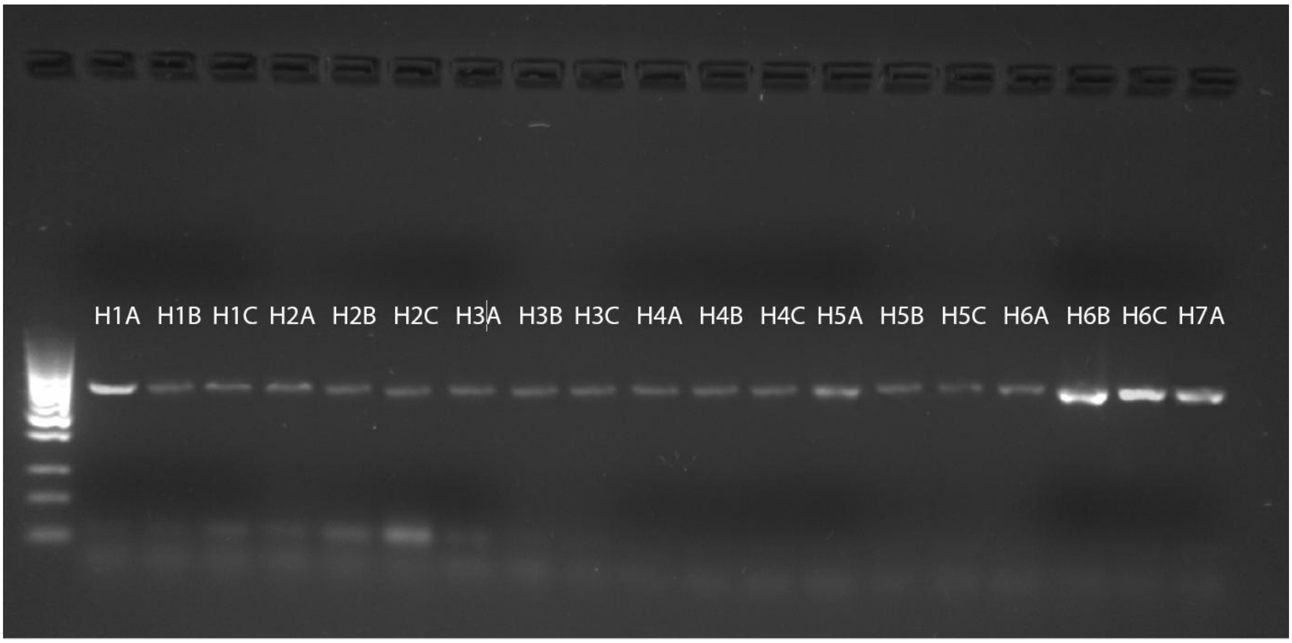

GEL 2

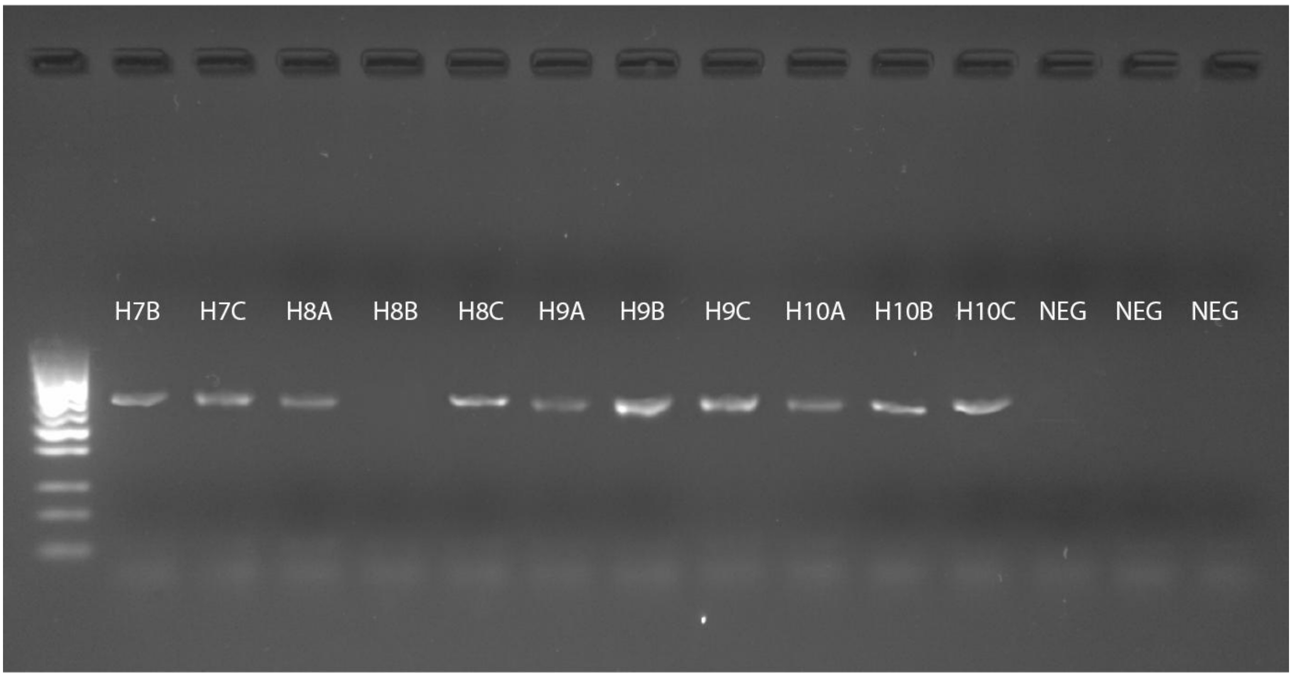

Supplement: Supplementary file 1 [file foods-15-03542-s001.zip › Supplementary File S2.pdf]
